# FGF23 promotes myocardial fibrosis in mice through activation of β-catenin

**DOI:** 10.18632/oncotarget.11623

**Published:** 2016-08-25

**Authors:** Huixin Hao, Xixian Li, Qingman Li, Hairuo Lin, Zhenhuan Chen, Jiahe Xie, Wanling Xuan, Wangjun Liao, Jianping Bin, Xiaobo Huang, Masafumi Kitakaze, Yulin Liao

**Affiliations:** ^1^ State Key Laboratory of Organ Failure Research, Department of Cardiology, Nanfang Hospital, Southern Medical University, Guangzhou, China; ^2^ Department of Oncology, Nanfang Hospital, Southern Medical University, Guangzhou, China; ^3^ Cardiovascular Division of the Department of Medicine, National Cerebral and Cardiovascular Center, Fujishirodai, Suita, Osaka, Japan

**Keywords:** fibroblast growth factor 23, β-catenin, TGF-β, myocardial fibrosis, ischemia/reperfusion, Pathology Section

## Abstract

Fibroblast growth factor 23 (FGF23) has been reported to induce left ventricular hypertrophy, but it remains unclear whether FGF23 plays a role in cardiac fibrosis. This study is attempted to investigate the role of FGF23 in post-infarct myocardial fibrosis in mice. We noted that myocardial and plasma FGF23 and FGF receptor 4 were increased in mice with heart failure as well as in cultured adult mouse cardiac fibroblasts (AMCFs) exposed to angiotensin II, phenylephrine, soluble fractalkine. Recombinant FGF23 protein increased active β-catenin , procollagen I and procollagen III expression in cultured AMCFs. Furthermore, intra-myocardial injection of adeno-associated virus-FGF23 in mice significantly increased left ventricular end-diastolic pressure and myocardial fibrosis, and markedly upregulated active β-catenin, transforming growth factor β (TGF-β), procollagen I and procollagen III in both myocardial infarction (MI) and ischemia/reperfusion (IR) mice, while β-catenin inhibitor or silencing of β-catenin antagonized the FGF23-promoted myocardial fibrosis in vitro and in vivo. These findings indicate that FGF23 promotes myocardial fibrosis and exacerbates diastolic dysfunction induced by MI or IR, which is associated with the upregulation of active β-catenin and TGF-β.

## INTRODUCTION

Fibroblast growth factor 23 (FGF23) is a newly discovered endocrine hormone produced by osteoblasts/osteocytes in bone that acts on the kidney and parathyroid glands to regulate phosphate homeostasis and vitamin D metabolism. [1] In addition to its physiologic actions, high level of FGF23 has also been shown to exert pathologic effects. [2] A series of studies have shown that FGF23 is markedly elevated in patients with chronic kidney disease [3] as well as in the general population [4-6] and can directly induce left ventricular hypertrophy. [7-9] Recombinant FGF23 can directly cause pathological cardiac hypertrophy, [8] while the expression of FGF23 can be up-regulated by systemic inflammation in cardiac fibroblasts. [10] These findings hint that cardiac-derived FGF23 may have potential to mediate cardiac remodeling. However, it remains unknown whether FGF23 is able to influence cardiac diastolic function by exerting effects on myocardial fibroblast cells.

Inflammation might be another mechanism linking FGF23 to cardiovascular disease. [5, 10] Experimental data showed that FGF23 increases the production of inflammatory markers such as transforming growth factor β (TGF-β), [5, 11] an important cytokine to induce fibrosis. TGF-β can increase expression of collagen I/III by stimulating the synthesis and deposition of collagen, fibronectin, proteoglycan and other intercellular substances. Over-expression of TGF-β has been demonstrated to be a common pathway for different pathological factors leading to myocardial fibrosis [12-14]. Another pro-fibrotic gene, β-catenin has been demonstrated to have crosstalk with TGF-β, [11, 15, 16] which is complex and context-dependent, [16] while our previous study demonstrated that fractalkine (FKN) plays an important role in the promotion of myocardial fibrosis and cardiac remodeling *via* up-regulating matrix metallopeptidase 9, procollagen I and III, and TGF-β. [17, 18]

According to the aforementioned clues, we hypothesized that FGF23 would promote cardiac fibrosis mediated by β-catenin and TGF-β. To test this hypothesis, we provided both *in vitro* and *in vivo* evidence that overexpression of FGF23 promotes proliferation of cardiac fibroblasts and increases myocardial fibrosis in mice with ischemia/reperfusion (IR) or permanent myocardial infarction (MI) through activation of β-catenin and upregulation of TGF-β.

## RESULTS

### Cardiac FGF23 was upregulated in failing heart or in response to pathological stimulations

**Figure 1:**
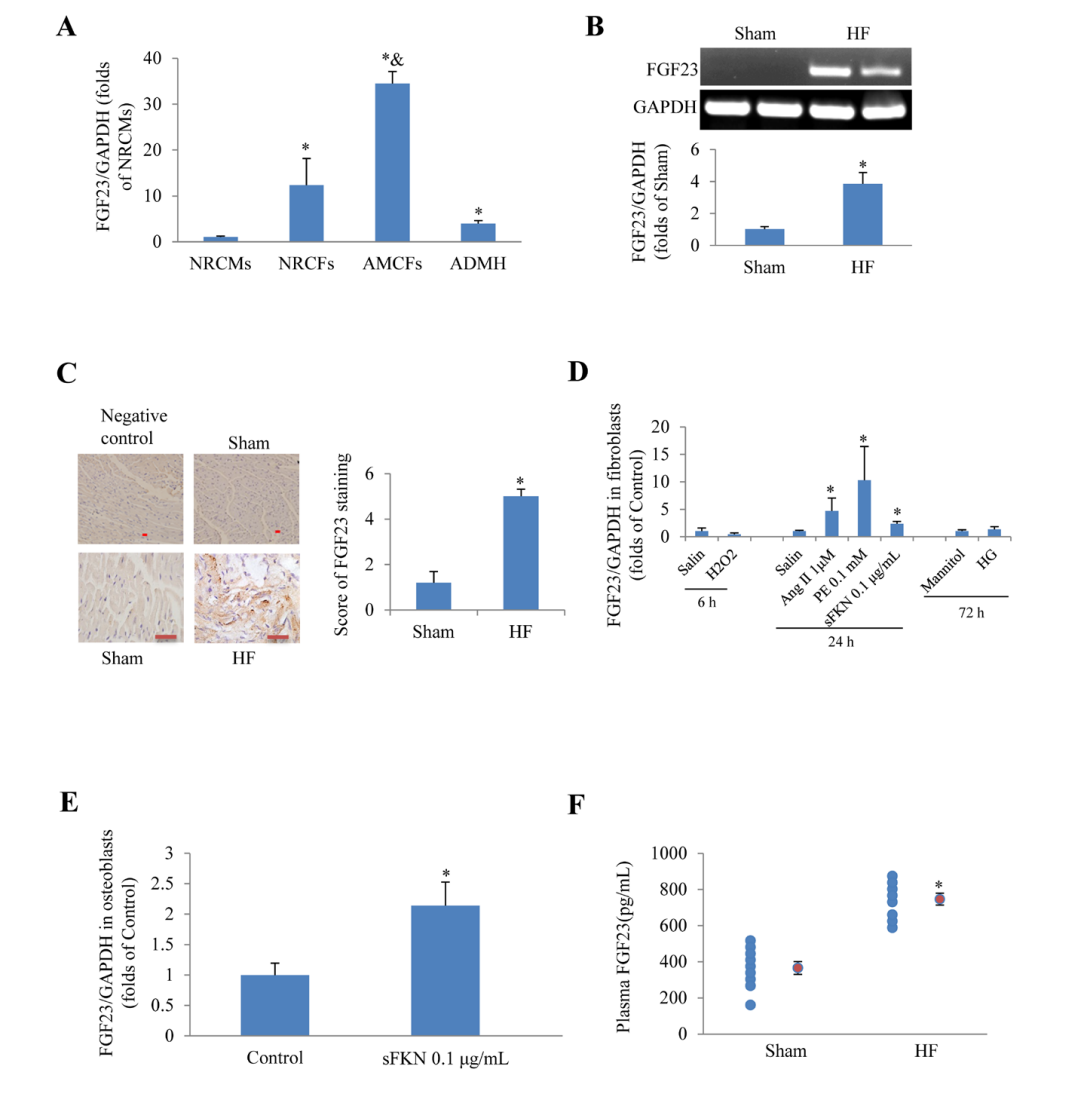
Expression of fibroblast growth factor 23 (FGF23) in rodent cardiac cells **A.** Detection of FGF23 mRNA expression in different murine cardiac cells, ^*^P < 0.05 *vs*. neonatal rat cardiomyocytes (NRCMs) group, ^&^*P* < 0.01 *vs*. neonatal rat cardiac fibroblasts (NRCFs) group. **B.** Myocardial FGF23 mRNA was upregulated in mice with heart failure (HF), ^*^P < 0.05 *vs*. Sham. **C.** Immunohistochemical detection of FGF23 expression in the mouse heart, scale bar = 10 *μ*m. **D.** Results of PCR for FGF23 in cultured adult mouse cardiac fibroblasts (AMCFs) in response to various stimulation for the indicated time (6, 24 or 72h), ^*^P < 0.05 *vs*. Control (saline or Mannitol). **E.** Expression changes of FGF23 in response to soluble fractalkine (sFKN) in cultured osteoblasts, ^*^P < 0.05 *vs*. Control. **F.** Circulating FGF23 concentrations in mice with post-myocardial infarction heart failure or sham operation (red circle refers mean of a group data), ^*^P < 0.05 *vs*. Sham. ADMH, Adult Mouse Heart; Ang II, Angiotensin II; PE, phenylephrine; HG, high concentration of glucose (25 mM).

First we tested whether FGF23 was expressed in cultured cardiac cells and whole heart. Real-time PCR revealed that FGF23 mRNA was significantly higher in neonatal rat cardiac fibroblasts, adult mouse cardiac fibroblasts (AMCFs) and adult murine heart than in neonatal rat cardiomyocytes, especially higher in AMCFs (Figure 1A). The basic levels of myocardial FGF23 mRNA and protein expression in mice were relatively low, but they were significantly up-regulated in heart failure induced by transverse aortic constriction (TAC) as evidenced by conventional PCR and immunochemistry (Figure 1B and 1C). Moreover, we noted that angiotensin II (Ang II), phenylephrine (PE) and soluble fractalkine (sFKN), stimulators known to induce cardiac fibrosis, also significantly increased the expression of FGF23 in cultured AMCFs (Figure 1D), while H_2_O_2_ and high glucose (HG) stimulation showed no difference from the control group (saline or mannitol). We also confirmed that sFKN stimulation upregulated FGF23 in osteoblasts (Figure 1E), in agreement with a postulation that FKN is increased in heart failure [17] and osteoblasts may be a source of circulating FGF23 in heart failure. Furthermore, we noted that plasma concentration of FGF23 measured using ELISA kit was significantly higher in mice with heart failure induced by MI than in sham group (Figure 1F). These findings indicate that FGF23 can be produced and upregulated in pathologic heart and AMCFs.

### FGF23 overexpression promoted fibroblast proliferation and upregulated fibrosis-related genes

**Figure 2:**
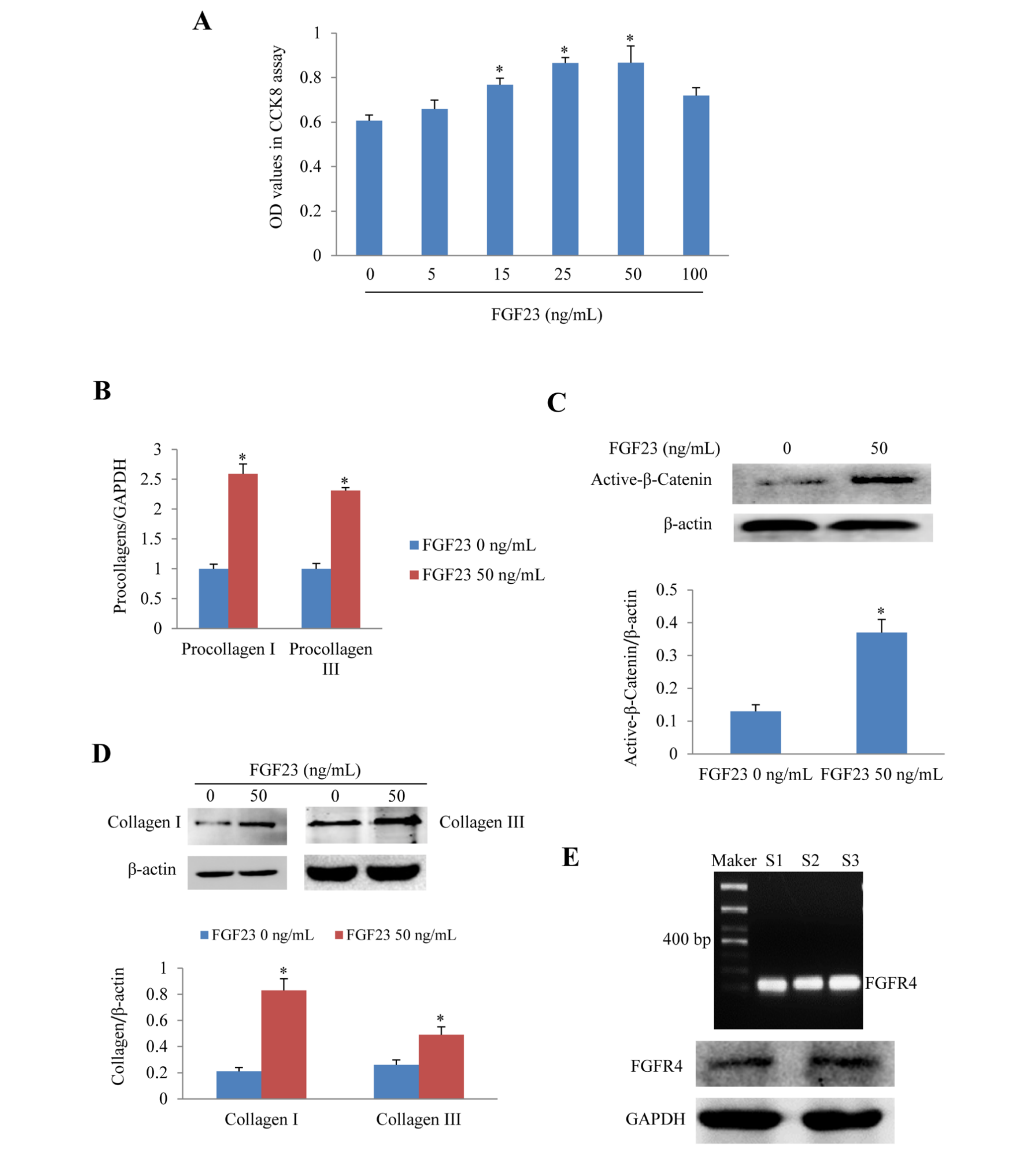
Effects of FGF23 stimulation on cultured adult mouse cardiac fibroblasts (AMCFs) **A.** Cell proliferation assay in AMCFs. **B.** Real-time PCR results of procollagen I/III expression **C.** Western blots of active β-catenin, **D.** Western blots of collagen I/III in AMCFs stimulated with FGF23 for 48 h. ^*^P < 0.05 *vs*. FGF23 0 ng/mL group, *n* = 8 in each group in panel A; *n* = 3-4 in other panels. **E.** Both Gene and protein of FGF receptor 4 (FGFR4) was expressed in cardiac fibroblasts isolated from adult mouse heart(S1-3: sham mice No 1-3).

We further investigated the effects of recombinant FGF23 protein on cultured AMCFs. CCK8 assay showed that FGF23 promoted AMCFs proliferation in a dose-dependent fashion. The addition of 50 ng/mL of FGF23 significantly increased proliferation (*P* < 0.05), whereas FGF23 did not further increase proliferation of fibroblasts under the concentrations of 100 ng/mL (Figure 2A). Then we performed the following experiments with FGF23 50 ng/mL. Real-time PCR showed that procollagen I/III gene expression was markedly increased in AMCFs exposed to FGF23 (Figure 2B), suggesting that high levels of FGF23 might promote AMCFs fibrosis. Western blot demonstrated that activation of β-catenin was enhanced in AMCFs by FGF23 stimulation (Figure 2C) and collagen I/III protein levels were also markedly increased when compared with the control group (Figure 2D). These findings reveal that FGF23 might promote fibrosis mediated by active-β catenin. Moreover, we found that gene and protein of fibroblast growth factor receptor 4 (FGFR4), a receptor of FGF23 which can exert biological role independent of coreceptor klotho in cardiomyocytes, [19] was also expressed in adult mouse cardiac fibroblasts (Figure 2E).

### Cardiac dysfunction induced by MI or IR was exacerbated in mice with FGF23-overexpression

**Figure 3:**
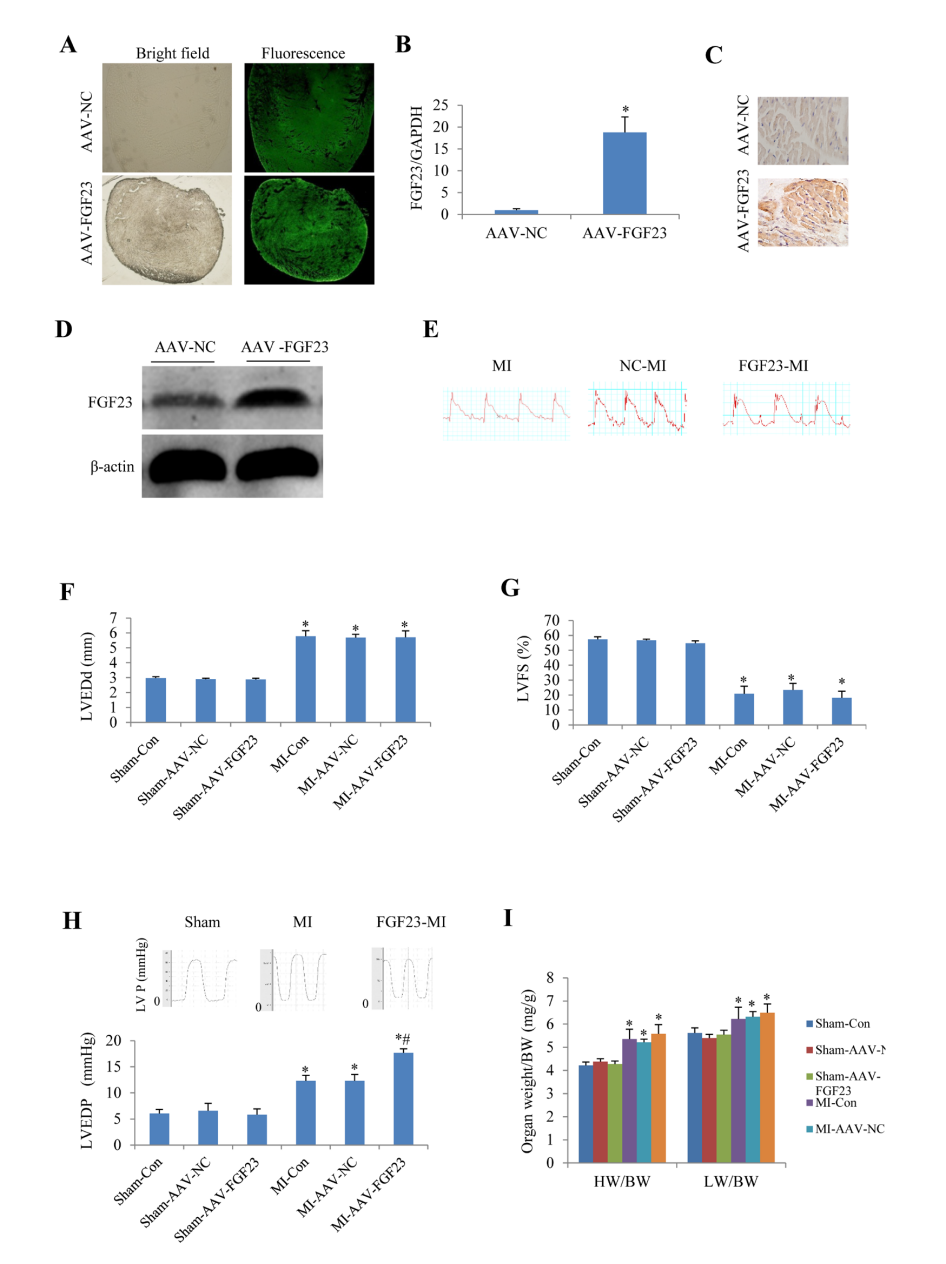
Effects of myocardial injection of AAV-FGF23 in mice with myocardial infarction (MI) on cardiac function **A.** Representative fluorescence microscopic pictures showing the infective efficiency of adeno-associate virus carrying short hairpin RNA targeting FGF23 (AAV-FGF23) in mouse heart detected by the green fluorescence of co-expressed EGFP at 4 weeks after intra-myocardial injection of AAV-FGF23 or AAV-negative control (NC). **B.** Real-time PCR of FGF23 overexpression (^*^P < 0.05 *vs*. NC). **C.** Immunohistochemistry of FGF23 expression. **D.** Western blot analysis of FGF23 overexpression. **E.** Representative electrocardiogram showing the similar ST-segment elevation in various MI groups. Effect of FGF23 overexpression on MI-induced cardiac remodeling were evaluated: **F.** Left ventricular end-diastolic diameter (LVEDd), **G.** Left ventricular fractional shortening (LVFS). **H.** Hemodynamic analysis of left ventricular end-diastolic pressure (LVEDP). **I.** Heart weigh/body weight ratio (HW/BW) in MI or Sham mice treated with AAV-NC or AAV-FGF23. ^*^P < 0.05 *vs*. the corresponding Sham group, ^#^*P* < 0.05 *vs*. MI- AAV-NC group, *n* = 6 in each group. NC: negative control; Con: control.

**Figure 4:**
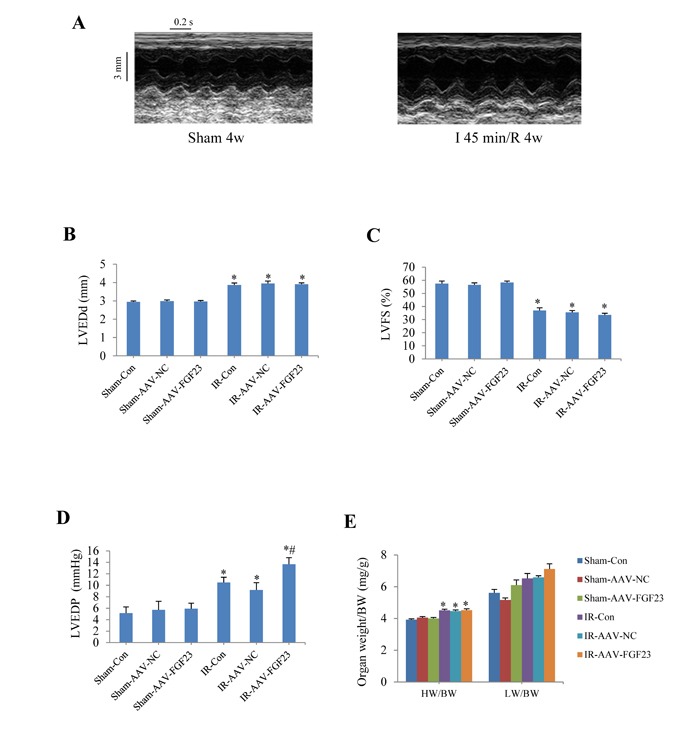
Effects of myocardial injection of AAV-FGF23 in mice with ischemia/reperfusion (IR) on cardiac function **A.** Representative M-mode echocardiographic images at 4 weeks after IR. **B.** Left ventricular end-diastolic diameter (LVEDd). **C.** Left ventricular fractional shortening (LVFS). **D.** Hemodynamic analysis of left ventricular end-diastolic pressure (LVEDP). **E.** Heart to body weight ratio (HW/BW) in IR or Sham mice treated with AAV-scramble or AAV-FGF23. ^*^P < 0.05 *vs*. the corresponding Sham group, ^#^*P* < 0.05 *vs*. IR-AAV-NC group, *n* = 6 in each group.

To investigate the role of FGF23 *in vivo*, cardiac overexpression of FGF23 was achieved by intra-myocardial injection of AAV-FGF23(adeno-associated virus carrying FGF23 gene) in 4 weeks old mice. Four weeks later, about 40% infection efficiency was obtained for the whole heart manifested by green fluorescence in cardiomyocytes under fluorescence microscopy as well as a significantly higher FGF23 expression level than in AAV-NC (negative control) group confirmed by real-time PCR, immunohistochemistry and western blot (Figure 3A-3D). Then mice were subjected to MI, IR or sham surgery. There was clear ST segment elevation after left coronary artery ligation in all the MI mice (Figure 3E). Echocardiography showed that MI mice had larger left ventricular end-diastolic diameter and smaller left ventricular fractional shortening than the sham ones, but there was no difference among the 3 MI groups (Figure 3F and 3G). While left ventricular hemodynamics showed that left ventricular end-diastolic pressure was significantly higher in AAV-FGF23-MI mice than in AAV-NC-MI mice (Figure 3H), suggesting that FGF23 overexpression mainly exacerbates left ventricular diastolic dysfunction. The heart weight/body weight ratio (HW/BW) and lung weight/body weight ratio (LW/BW) were larger in the MI groups than in their corresponding sham groups, but no significant difference was found among the 3 MI groups (Figure 3I). Similar results were obtained in IR mice (Figure 4), which further indicates an exacerbation in diastolic dysfunction by overexpression of FGF23.

### Overexpression of FGF23 *in vivo* promoted myocardial fibrosis induced by MI or IR

**Figure 5:**
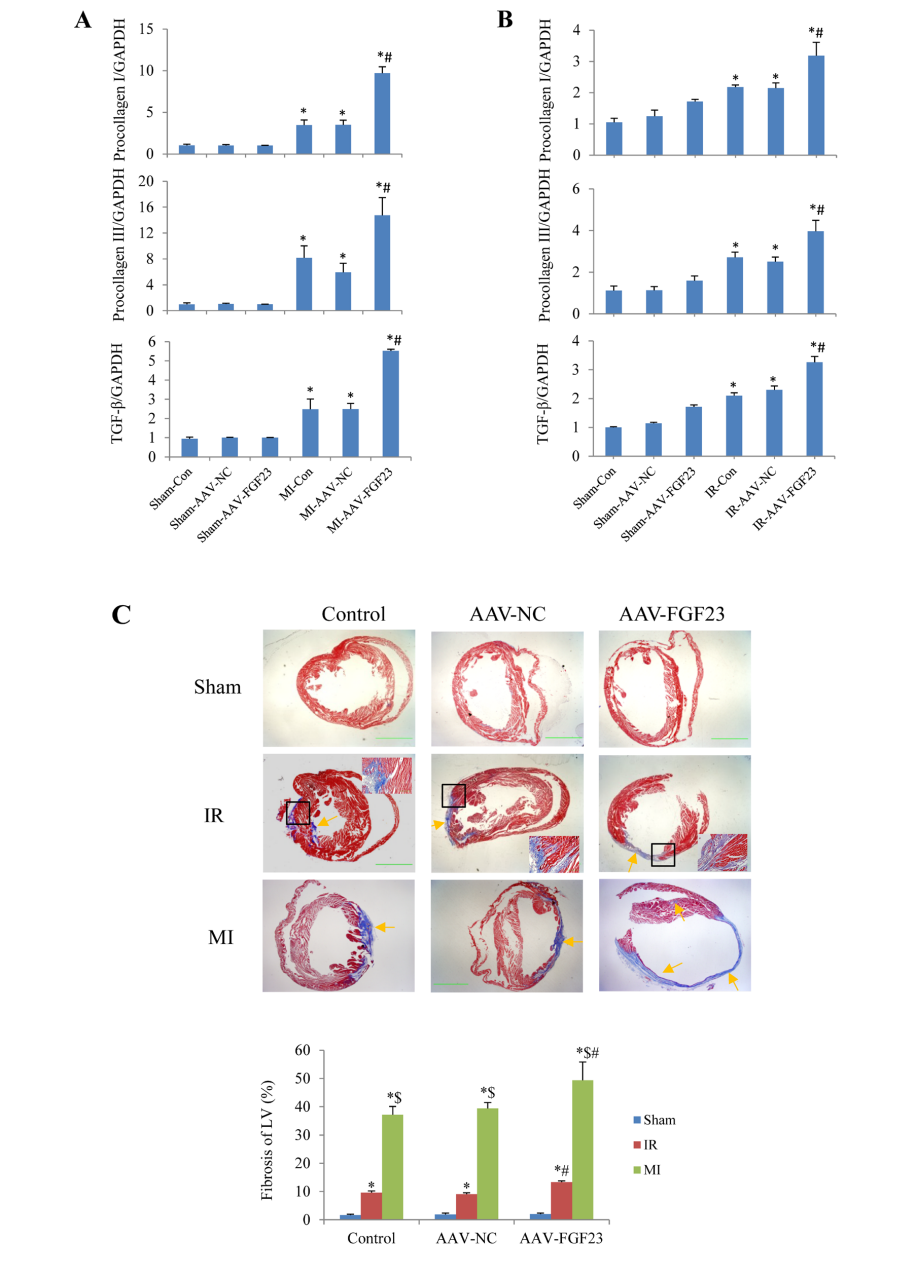
Effects of FGF23 on myocardial fibrosis induced by myocardial infarction (MI) or ischemia/reperfusion (IR) **A.**Real time-PCR analysis of TGF-β, procollagen I/III expression in MI or IR **B.** Real time-PCR analysis of TGF-β, procollagen I/III expression in IR mice. **C.** Representative pictures and quantitation of myocardial fibrosis with Masson's trichrome stain in RI and MI mice, scale bar = 1 mm. The inserted pictures were magnified from the black frames in the middle panels (scale bar = 200 *μ*m). ^*^P < 0.05 *vs*. the corresponding Sham group, ^$^*P* < 0.05 *vs*. the corresponding IR group; ^#^*P* < 0.05 *vs*. the corresponding MI or IR NC (negative control) group, *n* = 5 in each group.

Aa TGF-β is a recognized marker of myocardial fibrosis, we tested TGF-β expression levels in mice with MI for 4 weeks. Real-time PCR revealed that TGF-β mRNA level was significantly increased in AAV-FGF23-MI mice compared with AAV-NC-MI mice. What's more, the fibrosis related genes procollagen I/III were also markedly upregulated in FGF23 overexpressed MI mice (Figure 5A). Similar results were detected in IR mice (Figure 5B). In addition, myocardial fibrosis (fibrotic area/LV area) was markedly larger in AAV-FGF23-IR or MI mice than in AAV-NC-IR or MI mice (Figure 5C). Collectively, these data indicate that FGF23 promotes myocardial fibrosis induced by MI or IR through upregulation of TGF-β, procollagen I and III mRNA levels.

### FGF23 exerted effects on β-catenin activity and collagen I/III expression

**Figure 6:**
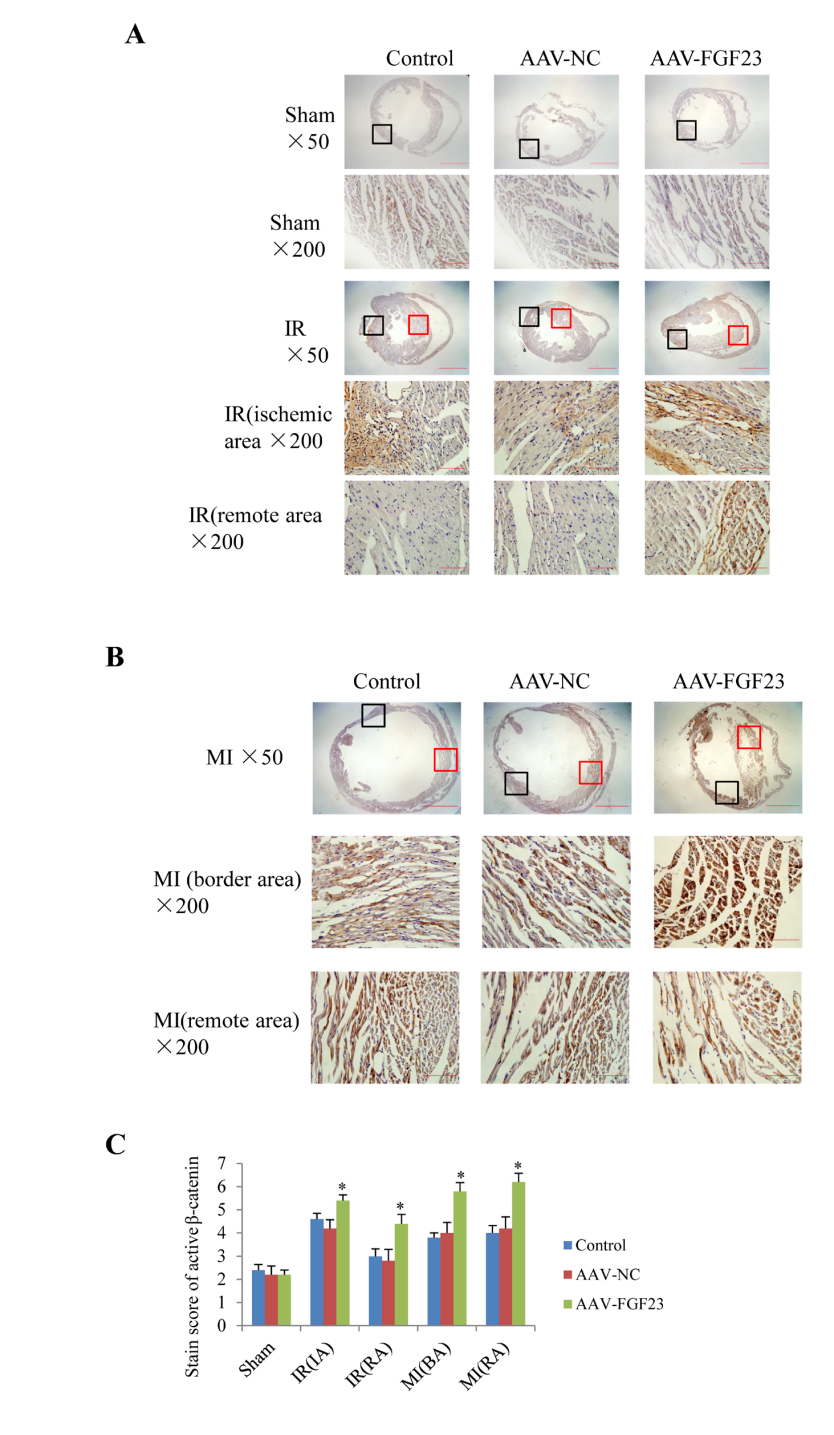
Immunohistochemical detection of myocardial active β-catenin **A.** Active β-catenin in heart of sham or ischemia/reperfusion (IR) mice treated with vehicle (control), AAV-NC or AAV-FGF23. Both the ischemic area (the black frames) and the remote area (the red frames) were magnified in lower panels. **B.** Active β-catenin in heart of mice with myocardial infarction (MI) treated with vehicle (control), AAV-NC or AAV-FGF23. Both the border area (the black frames) and the remote area (the red frames) were magnified in lower panels. **C.** Semi-quantitation of active β-catenin using a scoring system. IA, ischemic area; RA, remote area; BA, border area. ^*^P < 0.05 *vs*. AAV-NC group, *n* = 5 in each group. Scale bar = 1 mm and 200 *μ*m in pictures with ×50 and ×200 magnifications, respectively.

**Figure 7:**
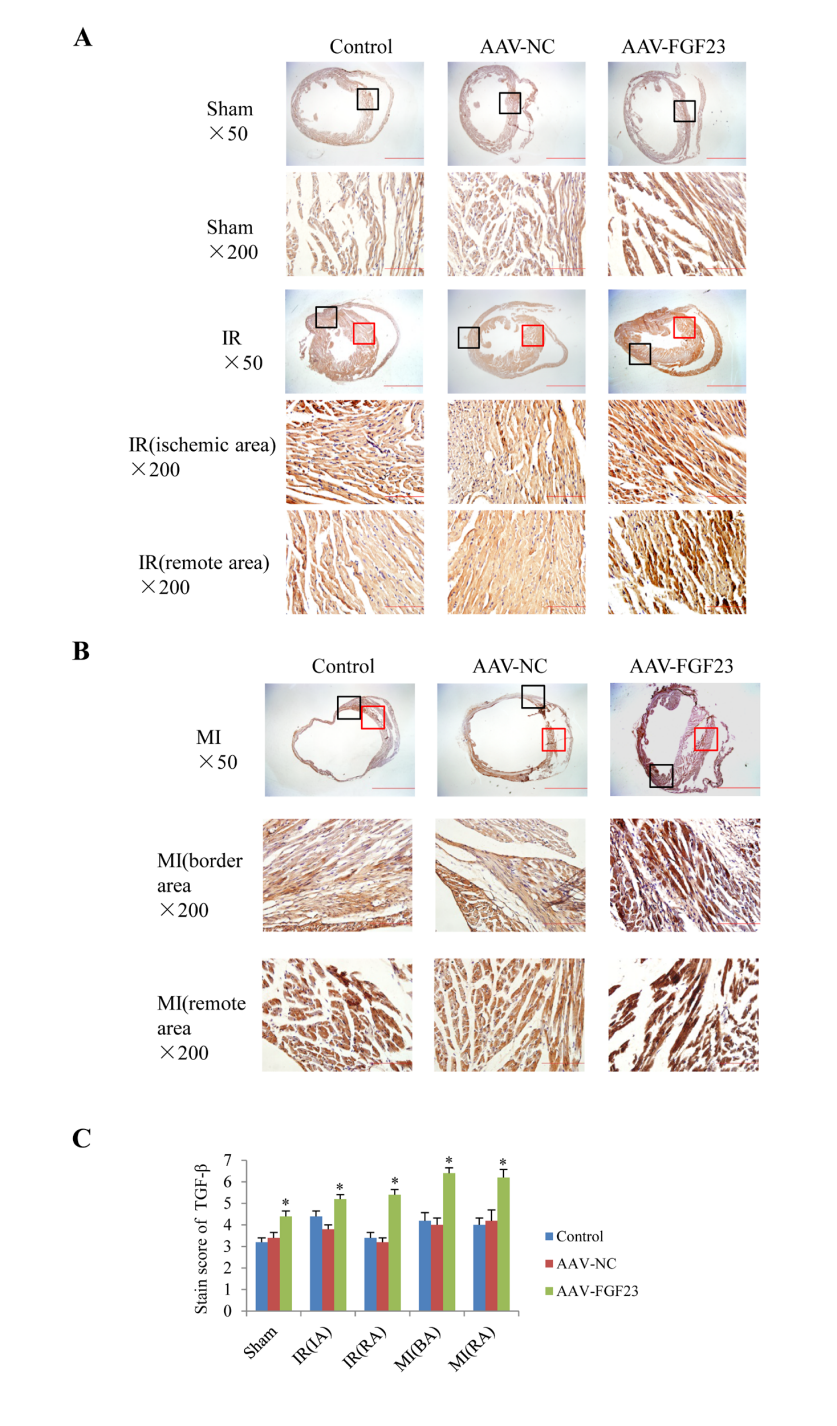
Effect of FGF23 overexpression on myocardial TGF-β expression **A.** Active TGF-β in heart of sham or ischemia/reperfusion (IR) mice treated with vehicle (control), AAV-NC or AAV-FGF23. Both the ischemic area (the black frames) and the remote area (the red frames) were magnified in lower panels. **B.** TGF-β in heart of mice with myocardial infarction (MI) treated with vehicle (control), AAV-NC or AAV-FGF23. Both the border area (the black frames) and the remote area (the red frames) were magnified in lower panels. **C.** Semi-quantitation of TGF-β using a scoring system. IA, ischemic area; RA, remote area; BA, border area. ^*^P < 0.05 *vs*. AAV-NC group, *n* = 5 in each group. Scale bar = 1 mm and 200 *μ*m in pictures with ×50 and ×200 magnifications, respectively.

**Figure 8:**
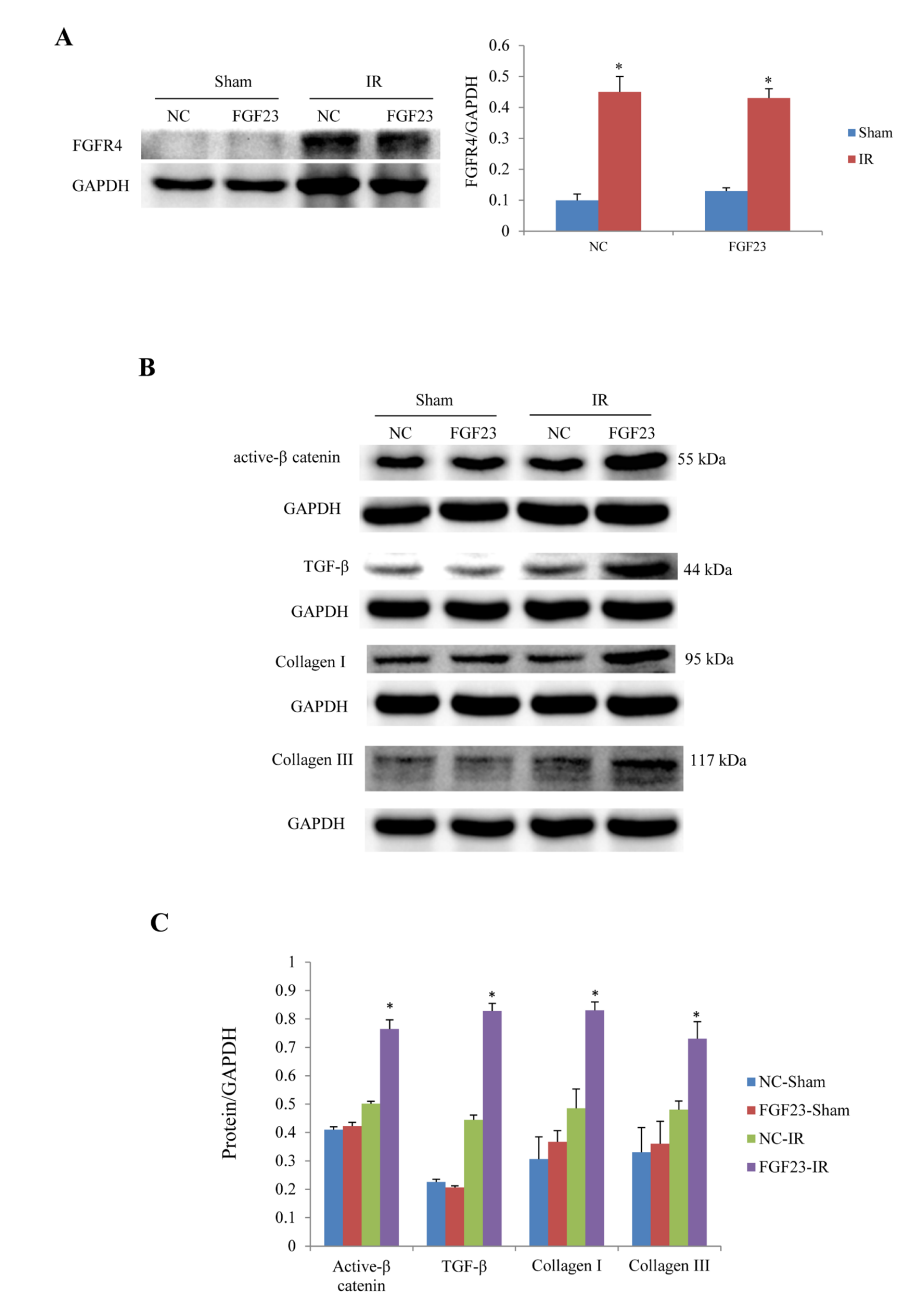
Western blot analysis of active β-catenin, TGF-β and collagen I/III expression in the heart of IR and sham mice treated with AAV-NC or AAV-FGF23 **A.** Western blot of FGFR4 (fibroblast growth factor receptor 4). **B.** Representative western blots of active β-catenin, TGF-β, collagen I and III. **C.** Semi-quantitative analysis of expression of those four proteins in panel B.^*^P < 0.05 *vs*. the corresponding negative group (NC)-sham group, *n* = 5 in each group.

In order to clarify the mechanism of FGF23 promoting myocardial fibrosis, we examined the changes of fibrosis-related molecules by Western blotting and Immunohistochemistry. Immunohistochemistry results clearly showed an increased expression of active β-catenin protein in AAV-FGF23-IR or MI mice than in AAV-NC-IR or MI mice (Figure 6A-6C), especially in the ischemic area of IR mice (Figure 6A and 6C) or border area of MI mice (Figure 6B and 6C). We further examined the effect of FGF23 on cardiac expression of TGF-β in IR or MI mice. Immunohistochemistry showed stronger immunoreactivity for TGF-β in FGF23 overexpression group than in the control group (Figure 7A-7C). Immunohistochemistry data were verified with Western blot, which confirmed significant increase of FGFR4, active β-catenin, TGF-β, collagen I and III in the heart of IR mice with FGF23 overexpression (Figure 8A-8C).

### Inhibition of β-catenin antagonized profibrotic role of FGF23

**Figure 9:**
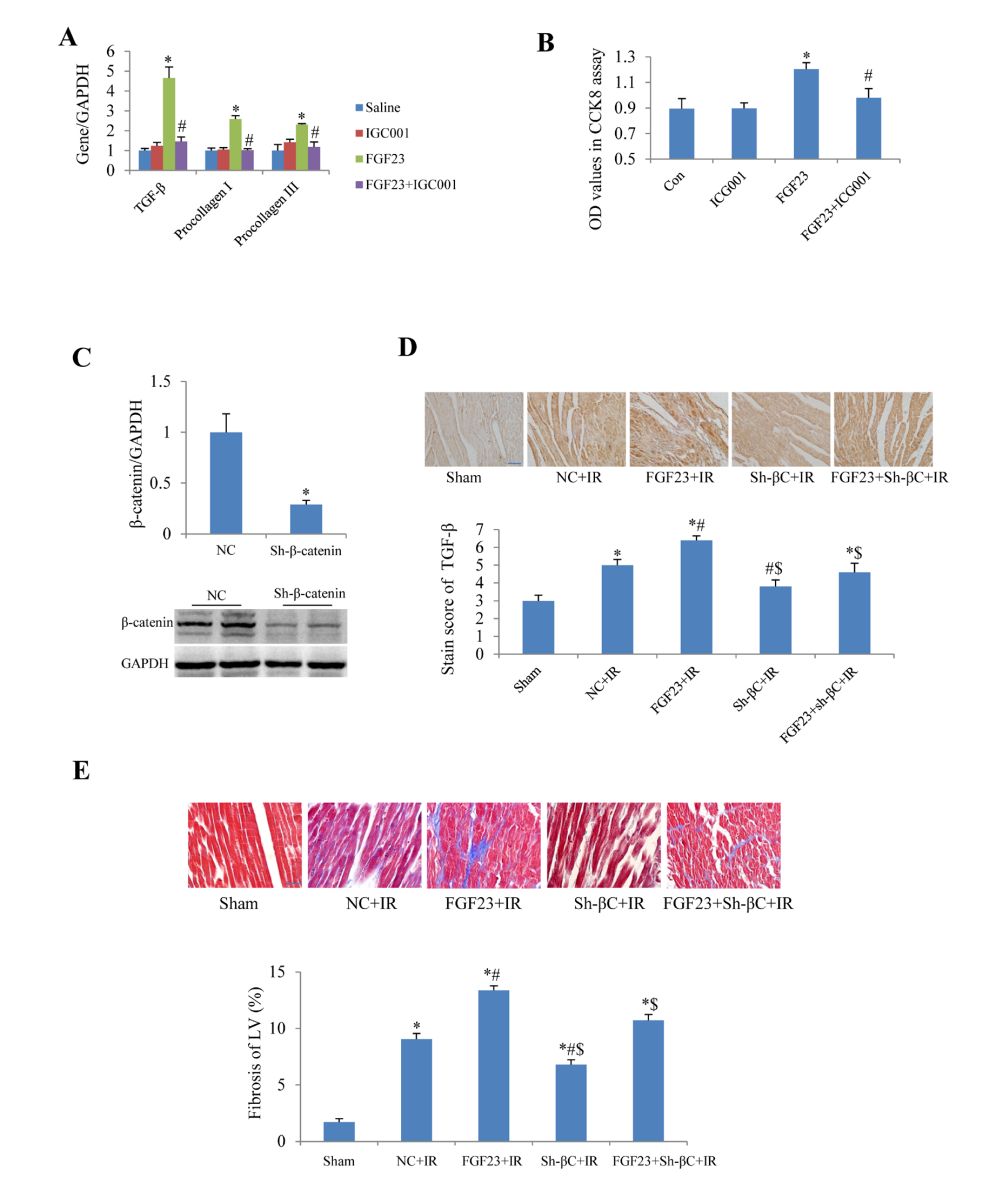
Effect of β-catenin inhibition on the fibrogenesis of FGF23 **A.** Real-time PCR results of TGF-β, collagen I and III in cultured adult mouse cardiac fibroblasts. **B.** Fibroblast proliferation measured with CCK8 assay. **C.** Silencing effects of β-catenin using adeno-associated virus (AAV) short hairpin RNA targeting β-catenin (sh-β-catenin or sh-βC), which were measured with real-time PCR and western blot. **D.** Semi-quantitation of TGF-β expression in heart using a staining scoring system. **E.** Representative pictures and quantitation of myocardial fibrosis with Masson's trichrome stain. Scale bar, 50 μM. ^*^P < 0.05 *vs*. the corresponding control group (saline in panel A, NC in panel C, sham in panel D and E), *n* = 5-8 in each group; ^#^*P* < 0.05 *vs*. FGF23 group in panel A and B or *vs*. NC+IR in panel D and E; ^$^*P* < 0.05 *vs*. FGF23+IR in panel D and E. FGF23, AAV-FGF23; IGC001, inhibitor of β-catenin (10 μM); Con, control; NC, negative control (AAV-NC); IR, ischemia/reperfusion.

In cultured adult mouse cardiac fibroblasts, IGC001, an inhibitor of β-catenin, significantly blocked the FGF23-induced upregulation of TGF-β, collagen I and III (Figure 9A). IGC001 also markedly inhibited the proliferation of fibroblasts as confirmed by CCK8 assay (Figure 9B). By silencing β-catenin using AAV-sh-β-catenin in the heart of mice (Figure 9C), we found that AAV-sh-β-catenin partially abolished IR-induced increase of TGF-β as well as FGF23 overexpression-enhanced TGF-β in the heart of IR mice (Figure 9D). Similarly, AAV-sh-β-catenin also attenuated FGF23-enhanced myocardial fibrosis in mice with IR (Figure 9E).

## DISCUSSION

Higher levels of FGF23 have been reported to be associated with cardiovascular disease [20-22] such as LV hypertrophy in both human [9] and experimental studies. [8] But the role of FGF23 in myocardial fibrosis and the potential mechanisms are not yet entirely elucidated. Myocardial fibrosis is presumed to play an important role in cardiac hypertrophy and accounts mainly for the development of left ventricular diastolic dysfunction. [23, 24] In the current study, we showed that FGF23 was upregulated in the failing heart. Hence, we aimed to investigate whether FGF23 exerts a direct effect on myocardial fibrosis and then further influences diastolic dysfunction. Interestingly, we found that myocardial FGF23 overexpression promotes myocardial fibrosis both *in vitro* and *in vivo*, and these detrimental effects were associated with the accumulation of TGF-β, collagen I and III mediated by the activation of β-catenin.

We demonstrated that FGF23 exposure could enhance AMCFs proliferation and increase the expression levels of fibrosis related genes such as TGF-β, procollagen I and III. Considering that compensatory hypertrophy and fibrosis in the non-infarct area is more common in MI model than in IR model, while pathological or therapeutical effect on fibrosis in ischemic area is easier to observe in IR model, we employed both MI and IR models to investigate the role of FGF23 on post-MI fibrosis. We noted that in the AAV-FGF23 infected mice, myocardial fibrosis induced by MI or IR was increased significantly compared with the corresponding control groups. These results indicate that overexpression of FGF23 promotes fibrosis in both AMCFs and MI/IR mice. It is unclear why FGF23 did not exert profibrotic effect in sham mice, the upregulated FGFR4 in response to myocardial ischemia may be attributable. Although it is common that many bioactive substances exert their role in response to pathological stimuli rather than physiological state, [25]it is need in the future study to clarify the mechanism of ischemia or reperfusion stimuli facilitating FGF23 to exert its profibrotic role.

β-catenin is a subunit of the cell surface cadherin protein complex and can be activated by multiple pathogenic cues. Increasing evidence suggests that sustained activation of Wnt/β-catenin is associated with the pathogenesis of fibrotic disorders such as lung fibrosis, liver fibrosis, skin fibrosis and renal fibrosis. [15, 26] It is demonstrated that the activation of this signaling is reparative in acute kidney injury, but sustained activation is detrimental in chronic kidney disease. [26] Moreover, Duan et al [27] reported that a pro-fibrotic Wnt/β-catenin-dependent injury response is important for preserving cardiac function after acute cardiac injury by activating the cardiac fibroblasts. Therefore our findings are reasonable that sustained activation of β-catenin by excessive FGF23 promoted myocardial fibrosis in MI/IR mice.

Once activated, β-catenin results in stabilization and translocates into the nucleus, where it binds to T cell factor /lymphoid enhancer-binding factor to stimulate the transcription of target genes, including fibrosis-related gene expression. [15, 26] Several studies implicate that there is cross-talk between Wnt/β-catenin and TGF-β signaling: Wnt/β-catenin signaling combinatorically works with TGF-β signaling in the process of fibrosis, and TGF-β signaling can induce expression of Wnt/β-catenin superfamily members and vice versa. [11, 15, 16] Importantly, this cross-talk is complex and context-dependent, and may promote fibrogenesis through co-regulation of fibrogenic gene targets. [16] We found activation of β-catenin in AAV-FGF23-MI/IR mice, accompanied by upregulation of TGF-β and the deposition of collagen I/III, and these effects were antagonized by inhibition of β-catenin, suggesting that active β-catenin might promote myocardial fibrosis *via* TGF-β signaling in FGF23 overexpressed mice.

In other hand, Itoh et al [2] reviewed that FGF2 promotes cardiac hypertrophy and fibrosis through the activation of FGF receptor (FGFR). Marco et al [28] showed that blockading FGFR partially reversed cardiac hypertrophy and fibrosis in a classic animal model of chronic kidney disease. A recent study confirmed that FGF23 can bind FGFR4 in cardiomyocytes to enhance hypertrophy, [19] interestingly, our data in this study showing that FGFR4 is also expressed in cardiac fibroblasts and upregulated in response to myocardial ischemia. Accordingly, we presume FGF23 might also promote myocardial fibrosis in a FGFR-dependent manner which needs to be investigated in the future.

In the present study, we analyzed the association of FGF23 and myocardial fibrosis. To the best of our knowledge, this is the first study that focuses the direct effect of FGF23 on the pathophysiology of myocardial fibrosis. Herein, we demonstrated for the first time that FGF23 promotes myocardial fibrosis induced by MI/IR, thus indicating that suppression of FGF23 signaling would be an effective approach to improve myocardial fibrosis.

## MATERIALS AND METHODS

All procedures were performed in accordance with our institutional guidelines for animal research that conforms to the Guide for the Care and Use of Laboratory Animals (NIH Publication, 8th Edition, 2011).

### Cell culture

The neonatal Sprague-Dawley (SD) rats at 1-3 days after birth were anaesthetized by 2% isoflurane inhalation. Isolation and culture of ventricular cardiomyocytes and fibroblasts was performed as described previously [18, 29, 30]. Osteoblast cells (MC3T3-E1) were obtained from the Cell Bank of the Chinese Academy of Sciences (Shanghai, China). Adult mouse cardiac fibroblasts (AMCFs) were isolated from adult mice and cultured using combined trypsin-type II collagenase digestion method.

To test FGF23 expression in different pathological conditions, cells were cultured for 4 days and then treated with 1 μM angiotensin II or 0.1 mM phenylephrine, or recombinant mouse soluble fractalkine (sFKN) (0.1 μg/mL, Chemokine domain, R&D, Minneapolis, MN) for 24 h, or 0.1 mM H_2_O_2_ for 6 h, or 25 M glucose for 72 h. AMCFs were treated with various concentrations (0, 5, 15, 25, 50 and 100 ng/mL) of recombinant FGF23 protein (26.1 kDa, 2629-FG-025, R&D Company).

### AMCFs identification and proliferation assay

Cardiac fibroblasts cultured on glass-bottomed dishes were fixed with 4% paraformaldehyde and permeabilized with 0.01% Triton X-100. After being blocked in 3% BSA, cells were incubated with the primary antibody (ab92547, Abcam Company) overnight at 4 °C. Cell nuclei were stained with DAPI. The fluorescence images were obtained using a Nikon confocal microscope.

Fibroblasts proliferation was determined by CCK8 assay according to the corresponding manufacturer's instructions and previous report [31]. In brief, fibroblast cells (1×10^5^ cells per well) were sub-cultured into 96-well plates with 200 μl of the complete culture medium. The next day, recombinant FGF23 proteins in different concentrations range (0 to 100 ng/ml) were added with serum free medium, and the medium was changed after 24 hours. Finally, the supernatant was removed, and 100 μl of DMEM/F12 medium containing 10 μl of CCK8 (Dojindo, China) was added to each well and incubation for 4 h at 37 °C. The absorbance values were read at 450 nm.

### Animal models of myocardial infarction and ischemia/reperfusion

Mice were kept at standard housing conditions with a light/dark cycle of 12 h and free access to food and water. C57BL/6 male mice (aged 8 weeks, weighing 20-25 g) were intraperitoneally anesthetized with a mixture of xylazine (5 mg/kg, intraperitoneal) and ketamine (100 mg/kg, intraperitoneal), and the depth of anesthesia was monitored from the disappearance of pedal withdrawal reflex. Mice were then subjected to a left-sided thoracotomy and the left coronary artery ligation to induce myocardial infarction (MI), or ligation for 45 min followed by 4 weeks of reperfusion to induce ischemia/reperfusion (IR) as described elsewhere. [32-34] Ischemia was judged by myocardial blanching and electrocardiogram ST-segment elevation. Sham operated mice underwent the same procedure without ligation of left coronary artery. Four weeks after the operation, mice were killed by an overdose of pentobarbital sodium (150 mg·kg^−1^, i.p.), and cervical dislocation, and their hearts were extracted for further analysis. For histological examinations, hearts were fixed in 10% formalin, whereas for molecular analysis the hearts were snap-frozen in liquid nitrogen and stored at −80°C until used.

### Construction and infection of recombinant AAV-FGF23 and AAV-sh-β-catenin

pAAV2/9-CMV-ZsGreen (AAV: adeno-associate virus) vectors carrying FGF23 (NM_022657, 756bp) or short hairpin of β-catenin (sh-β-catenin) (Ctnnb1, NM_007614.3, 3640bp) or negative control were generated by a professional company (Vigene, Shandong, China). For *in vivo* infection, pAAV2/9-CMV-ZsGreen-FGF23 or sh-β-catenin or control virus particles (3.3 × 10^11^ pfu/ml) were administered by direct injection in the left ventricular free wall (2 sites, 10 μl/site) in mice at 4-weeks-old using a syringe with a 30-gauge needle, and four weeks later, sham, MI or I/R surgery was performed. [33] Transduction efficiency of *in vivo* gene transfer by AAV was assessed by EGFP fluorescence (510 nm) in cryosectioned heart slices using a fluorescence microscopy.

### Echocardiography

Non-invasive transthoracic echocardiography was performed in mice using a Sequoia 512 system with a 17L-5 probe (Siemens, Germany). [30]

### Invasive hemodynamic study

LV hemodynamics were evaluated before killing. [35] Mice from each group were anaesthetized with isoflurane inhalation at a concentration of 1.5% and were ventilated as mentioned above. A Millar catheter was inserted *via* the right carotid artery and carefully introduced into the left ventricle to measure left ventricular hemodynamic parameters.

### PCR assay

Total RNA was extracted from cultured cells and mouse heart tissues (total RNA isolation system, Omega, Norcross, GA, USA). Conventional or quantitative real-time PCR using a Quantitect SYBR Green RT-PCR kit (QIAGEN) and an Applied LightCycler 480 system targeting the genes of FGF23, TGF-β, procollagen I, procollagen III, fibroblast growth factor receptor 4 (FGFR4), β-actin and GAPDH, was performed (primer sequences were listed in Table 1).

**Table 1:** Sequences of primers for real-time PCR

Transcripts	Forward primer (5′–3′)	Reverse primer (5′–3′)	Product size (bp)
FGF23 (mouse)	GCACTGCTAGAGCCTATCC	ATGGCTCCTGTTATCACCAC	208
FGF23 (rat)	GATGGCCATGTAGACGGAAC	GGTAGTGATGCTTCGGTGAC	232
Procollagen I	CTCGTCACAGCCTTCAC	AATCCAGTAGTAATCGCTCTTC	176
Procollagen III	CTACACCTGCTCCTGTCATT	CCACCCATTCCTCCGACT	232
TGF-β(rat)	GGCGGTGCTCGCTTTGTA	GCGGGTGACTTCTTTGGC	141
FGFR4 (mouse) GAPDH (mouse)	GCGTGCAGTTTCTTCTCCAT ATGTGTCCGTCGTGGATCTGA	TCAATAACGGACCCCAAG TTGCTGTTGAAGTCGCAGGAG	109 151

### Western blot and measurement of plasma FGF23

The following antibodies were used for the Western blotting analysis: anti-FGF23 (Santa-Cruz), anti-active β-catenin and anti-β-catenin (Millipore), anti-TGF-β (Abcam), anti-collagen I or III (Bioss, Beijing, China), anti-FGFR4 (Abcam). Blotting of β-actin or GAPDH (ZSGB-Bio, Beijing, China) was used as a loading control.

Plasma FGF23 concentrations were measured using a mouse Enzyme-Linked Immunosorbent Assay kit (EZMFGF23-43K, Merck Millipore Corporation, Germany) according to the manufacturer's protocol instruction.

### Immunohistochemistry

The heart tissue sections were incubated with mouse anti-active β-catenin antibody (Millipore) or rabbit anti-TGF-β antibody (Abcam) overnight at 4°C. The staining intensity was scored as: 0 (negative), 1 (weak), 2 (medium) or 3 (strong). The extent of staining was scored as 0 (0%), 1 (1-25%), 2 (26-50%), 3 (51-75%) or 4 (76-100%), according to the percentages of positively stained areas in relation to the whole version field. The sum of the staining intensity and extent score was used as the final staining score (0-7) for β-catenin or TGF-β.

To evaluate the extent of cardiac fibrosis, each heart was cut into four sections and stained with Masson trichrome. NIH Image J software was used (6-10 randomly chosen sections per sample) for quantification of the myocardial fibrosis.

### Statistical analysis

Quantitative data were expressed as the mean ± SEM (standard error of mean). Statistical significance between two experimental groups was analyzed using Student's two-tailed *t*-test, while comparisons of parameters among ≥ 3 groups were analyzed by one-way or two-way ANOVA followed by Bonferroni's correction for post hoc multiple comparisons. In all analyses, *P* < 0.05 was considered to indicate statistical significance.
